# The Three Gorges Dam: Does the Flooding Time Determine the Distribution of Schistosome-Transmitting Snails in the Middle and Lower Reaches of the Yangtze River, China?

**DOI:** 10.3390/ijerph15071304

**Published:** 2018-06-21

**Authors:** Yu Yang, Sheng-Bang Zheng, Ya Yang, Wan-Ting Cheng, Xiang Pan, Qing-Qing Dai, Yue Chen, Lan Zhu, Qing-Wu Jiang, Yi-Biao Zhou

**Affiliations:** 1 Fudan University School of Public Health, Building 8, 130 Dong’an Road, Xuhui District, Shanghai 200032, China; 16211020071@fudan.deu.cn (Y.Y.); zheng9004@126.com (S.-B.Z.); yayang16@fudan.edu.cn (Y.Y.); 15211020042@fudan.edu.cn (W.-T.C.); 14211020063@fudan.edu.cn (X.P.); jiangqw@fudan.edu.cn (Q.-W.J.); 2 Key Laboratory of Public Health Safety, Fudan University, Ministry of Education, Building 8, 130 Dong An Road, Xuhui District, Shanghai 200032, China; 3 Fudan University Center for Tropical Disease Research, Building 8, 130 Dong’an Road, Xuhui District, Shanghai 200032, China; 4 Department of Statistics, Oklahoma State University, Stillwater, WA 74078, USA; qingqing.dai@okstate.edu (Q.-Q.D.); lan.zhu@okstate.edu (L.Z.); 5 School of Epidemiology and Public Health, Faculty of Medicine, University of Ottawa, 600 Peter Morand Crescent, Ottawa, ON K1G 5Z3, Canada; ychen@uottawa.ca

**Keywords:** Three Gorges Dam, *Schistosomiasis japonica*, *Oncomelania hupensis*, flooding, geographical distribution

## Abstract

*Background*: Schistosomiasis is one of the most devastating tropical diseases in the world. *Oncomelania hupensis* is the only intermediate host of *Schistosoma japonicum*, and its growth and development are sensitive to environmental factors. The Three Gorges Dam has substantially altered the water level in the Yangtze River. This study focused on the impact of the flooding time on the occurrence of *Oncomelania* snails in Hunan Province, China. *Methods*: The data regarding *Oncomelania* snails were collected from the Schistosomiasis Atlas of the People’s Republic of China. Air temperature, hours of daylight and relative humidity from 1995 to 2002 were collected from the China Meteorological Data Sharing Service System. The data for rainfall and days inundated with water were collected from the Hunan flood control information system and hydrological stations in Hunan Province. A generalized additive model was used to estimate the impact of these factors on the presence or absence of snails. *Results*: The number of days inundated with water in the areas with snails ranged from 56 to 212 days. However, 82 percent of the areas without snails were inundated with water less than 60 days. The lowest air temperature in a year in the areas without snails ranges from −2.88 °C to −2.10 °C, and the range was from −2.88 °C to −2.34 °C for areas with snails. Annual rainfall in the areas with snails ranged from 989 to 1565 mm, and the range was from 1230 mm to 1647 mm for the areas without snails. The results from the generalized additive model showed that the number of days inundated with water, lowest air temperature in a year, annual rainfall, days of daily rainfall greater than 0.1 mm, and hours of daylight were the factors that significantly affect the occurrence of snails in Hunan Province, China. *Conclusions*: The number of days inundated with water may be a key factor determining the geographical distribution of *Oncomelania* snails in Hunan Province and the favorable number of days inundated with water for the survival of snails ranges from about 2 to 7 months.

## 1. Introduction

**Figure 1:**
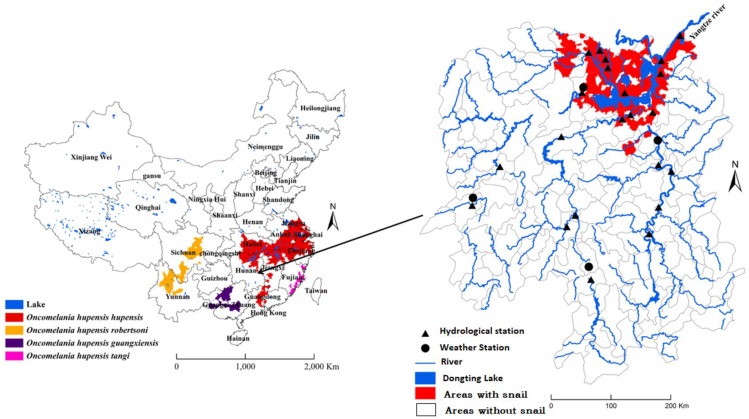
Geographical distribution of *O. hupensis* in Hunan Province, China.

Schistosomiasis remains a serious public health problem worldwide, affecting more than 200 million people in approximately 76 countries with a loss of 1.53 million disability-adjusted life years (DALYs) [1].The disease is caused mainly by three species of blood fluke genus *Schistosoma*: *S. japonicum*, *S. mansoni* and *S. haematobium*. Schistosomiasis japonica, which is caused by *S. japonicum*, is widely endemic in China and the Philippines. *Oncomelania hupensis*, the only intermediate host of *S. japonicum*, plays a vital role in *S. japonicum* transmission in China, which makes *Oncomelania* snails a crucial target for disease control. The distribution of *O. hupensis* largely depends on environmental conditions such as vegetation coverage, temperature, soil type, and water level [2].There are four subspecies of *O. hupensis* on the mainland of China with discrete patterns of distribution and they are *O. h. hupensis*, *O. h. robertsoni*, *O. h. Guangxiensis* and *O. h. tangi*. These snails are only found in 12 provinces located along the Yangtze River and southern China (Figure 1) [3,4,5]. It seems likely that features of the landscape have influenced and channeled the habitats of four subspecies of *O. hupensis.* The Three Gorges Dam (TGD), a world-class water conservancy project, is located in the upper reaches of the Yangtze River, and the middle and lower reaches of this River are the largest endemic area of schistosomiasis in China. The TGD began to impound water and sediment discharge in 2003, and its water level rose to 135 m by the end of 2003. Since 2009, the water level has been kept at 175 m throughout November and December and is lower in the other months [6].The TGD represents a significant land use change in topography and evaporation that is expected to result in changes in the regional weather and climate patterns [7]. Previous studies by the Chinese Meteorological Institute suggested that the TGD reservoir area would alter local patterns of precipitation, wind, and temperature, and estimated that the annual average near surface air temperature in the vicinity of the TDG would increase by 0.3 °C [8]. It has been previously reported that some large-scale hydro projects (e.g., the Sudanese Gezira-Managil Dam, the Egyptian Aswan High Dam and the Ethiopian Melkasadi Dam) have resulted in the emergence or re-emergence of schistosomiasis [9,10,11,12,13]. After the Three Gorges Dam started operating, various studies have reported on the dam’s impact on the distribution of *Oncomelania* snails. Most of these reports revealed that the TGD has contributed to a reduction in the density of *Oncomelania* snails and/or changes in the distribution of *Oncomelania* snails in the downstream areas of the dam, including the Dongting and Poyang Lakes [6,14,15,16,17]. *O. h. hupensis* is strictly distributed in the Yangtze River basin. These *Oncomelania* snails are amphibious, thriving on land during winter and in water during summer, and their growth and development are sensitive to environmental factors such as flooding, temperature, humidity and vegetation [18,19,20,21]. Water is a necessary condition for schistosomiasis transmission [22]. The TGD has altered the water level of the Yangtze River and largely controlled flooding, which may the reason for observed reductions in the prevalence of infection with *S. japonicum* and the density of *Oncomelania* snails in the downstream areas [23]. To determine whether flooding is a key or critical factor for the presence of *Oncomelania* snails in the middle and lower reaches of the Yangtze River, China, and under the condition of the controlling effect of rainfall and air temperature, we studied the impacts of flooding on the presence or absence of *O. h. hupensis* in Hunan Province before 2003 when the dam functioned. We also examined the critical time window of flooding for the survival of *Oncomelania* snails.

## 2. Methods

### 2.1. Study Area

Hunan Province, along the middle and lower reaches of the Yangtze River, is one of the schistosomiasis endemic areas in China. *Oncomelania* snails are mainly distributed in the vast floodplains of the Dongting Lake region, northeast of Hunan Province. The Dongting Lake, located at 28°30′–30°20′ N and 111°40′–113°40′ E, is the second largest fresh water lake in China. It has extensive connections with the Yangtze River in the north and also with four rivers (the Xiang, Zi, Yuan and Li Rivers) in the south and west. When the wet season begins in April or May, with the flows from rivers and the backflow from the Yangtze River, the water level rises, and many marshlands in Hunan Province become immersed with water until September or October. Grass-covered marshlands are suitable for cattle grazing and snails breeding, which makes the Dongting Lake region one of the regions most affected by schistosomiasis, historically.

### 2.2. Data Collection

#### Snail Distribution

*O. h. hupensis* was mainly distributed in the northeastern part of Hunan Province. The distribution of snails was marked using ArcGIS10.0 (Environmental Systems Research Institute, Inc., Redlands, CA, USA) with the village as the basic unit, according to the Schistosomiasis Atlas of the People’s Republic of China (Figure 1) [24], and the area distribution of *O. h. hupensis* was largely unchanged during the study period. Based on the presence or absence of *Oncomelania* snails, study areas in Hunan Province were divided into two types: areas with and without snails.

### 2.3. Environmental Factors

#### 2.3.1. Air Temperature

Air temperature was obtained from the China Meteorological Data Sharing Service System at four monitoring sites in the Hunan Province: Changde (111.51° E, 29.02° N), Changsha (112.59° E, 28.12° N), Zhijiang (109.78° E, 27.44° N) and Yongzhou (111.37° E, 26.13° N). Changde and Changshaare are located in areas with *Oncomelania* snails, while Zhijiang and Yongzhou belong to the areas without *Oncomelania* snails. The downloaded temperature data included monthly average temperature and annual lowest air temperature in the four sites from 1995 to 2002. Then, annual average temperature was calculated for these 8 years.

#### 2.3.2. Hours of Daylight and Relative Humidity

The data for daylight and relative humidity in Hunan Province were also obtained from the China Meteorological Data Sharing Service System. We downloaded monthly hours of sunshine and monthly relative humidity at four weather stations in Hunan Province for the period from 1995 to 2002. Then annual average hours of daylight and annual relative humidity over these 8 years were calculated.

#### 2.3.3. Rainfall

Daily rainfall and the number of days of daily rainfall greater than 0.1 mm were collected from the Hunan flood control information system at 15 selected monitoring sites, where 7 sites were located in the areas with snails and 8 sites were in areas without snails. The annual rainfall from 1996 to 2002 (data were not complete for the year of 1995) was calculated according to the daily rainfall.

#### 2.3.4. Number of Days Inundated with Water

**Figure 2:**
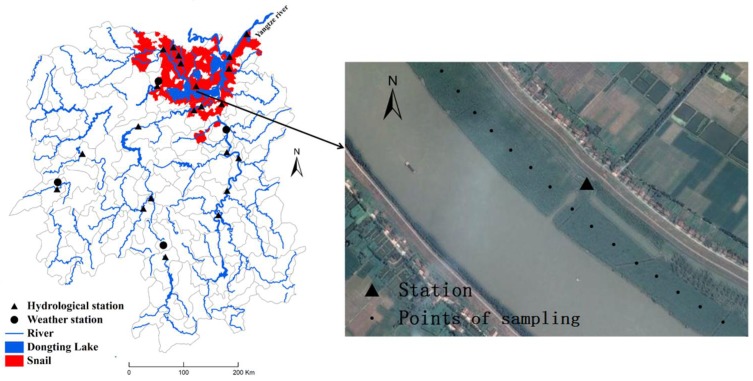
Systematic sampling of points along the marshland.

Daily water levels from 1995 to 2002 were obtained from 17 hydrological stations in Hunan Province. Among these stations, 9 were located in areas with snails and the other 8 were located in the areas without snails. A grassland close to each station and possibly suited to snails (so-called winter-land, summer-water breeding stimulation) was selected to estimate the number of days inundated with water. With the help of Google Earth, 20 points along the marshland were selected by systematic sampling (200 m apart between points) (Figure 2). The elevation of each point was obtained from the Hunan grid map of elevation. We calculated the flooding time per year by comparing daily water levels and the elevation of each point. If the water level in a day was higher than the elevation, this point was considered to be flooded, or otherwise, not flooded.

### 2.4. Statistical Analysis 

All data were entered in Microsoft Office Excel 2007 (Microsoft Corp., Redmond, WA, USA) and statistical analyses were done with IBM SPSS software (version 20.0, IBM, Armonk, NY, USA) and R (R version 3.3.0, R project, Vienna, Austria) The Shapiro-Wilk test was used to test the normal distribution of environmental data, including air temperature, hours of daylight, relative humidity, rainfall and days inundated with water. The Whitney U test was used to compare medians of the environmental factors. A generalized additive model was used to evaluate the impacts of rainfall, days inundated with water, air temperature, hours of day light and relative humidity on the occurrence or absence of snails. 

## 3. Results

### 3.1. Environmental Factors in Different Areas

**Figure 3:**
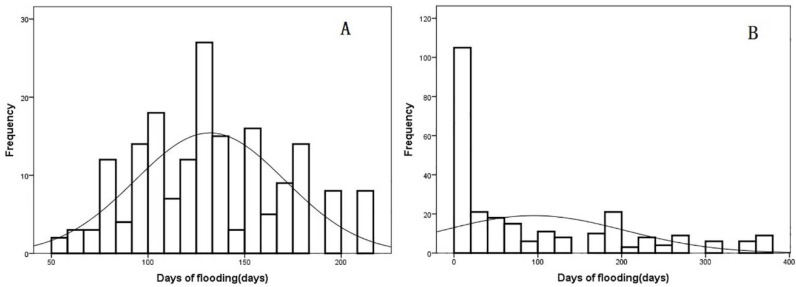
Histograms of the days inundated with water in the two areas. Number of days inundated with water in the areas with snails (**A**) and without snails (**B**).

As shown in Table 1, the ranges of temperature were wider in the areas without snails than in the areas with snails. However, the temperatures, including annual average temperature and lowest air temperature in a year, were not significantly different between the areas with and without snails (*p* > 0.05). The annual rainfall in the areas with snails ranged from 989 mm to 1565 mm, compared with the range from 1230 mm to 1647 mm for the areas without snails. The heaviest rainfall occurred in the areas without snails, which also has a higher altitude. The number of days inundated with water in the two areas were significantly different (*Z* = 6.849, *p* < 0.05). The number of days inundated with water in the areas with snails were normally distributed (*Z* = 1.315, *p* = 0.063). The number of days inundated with water in the areas with snails ranged from 56 to 212, and the median was 126 days. In the areas without snails, the distribution of the data did not follow normal distribution (*Z* = 3.419, *p* < 0.05); about 82 percent of the areas had less than 60 days inundated with water (Figure 3). The relative humidity (*Z* = −5.053, *p* < 0.05) and the hours of daylight (*Z* = −7.808, *p* < 0.05) were both significantly related to the occurrence of snails.

### 3.2. Generalized Additive Model (GAM) of the Distribution of Snails

The impact of factors on the occurrence of snails were analyzed by using a generalized additive model that allows non-linear relationships between response and explanatory variables. As shown in Table 2, the occurrence of snails was significantly related to the interaction between annual rainfall and lowest air temperature in a year (*Z* = −3.697, *p* < 0.05) as well as the interaction between lowest air temperature in a year and days of rainfall greater than 0.1 mm (*Z* = −3.540, *p* < 0.05). Factors including, days inundated with water (χ^2^ = 31.28, *p* < 0.05), lowest air temperature in a year (*Z* = 3.648, *p* < 0.05), annual rainfall (*Z* = −3.641, *p* < 0.05), days of daily rainfall greater than 0.1 mm (*Z* = −3.658, *p* < 0.05), and hours of daylight (*Z* = −2.674, *p* < 0.05), were also significantly associated with the occurrence of snails. The number of days inundated with water was related to the occurrence of snails (χ^2^ = 31.28, *p* < 0.05) but not in a linear fashion, as shown by the smooth function in the GAM. Relative humidity was highly correlated with hours of daylight with a correlation coefficient of 0.95. Therefore, with hours of daylight in the model, relative humidity was not significant and it was excluded from the final model.

## 4. Discussion

Environmental changes are important influencing factors for the growth and development of *Oncomelania* snails. The Three Gorges Dam project was approved in 1992, aiming to control flooding for the Yangtze River [25]. Since sluice gates of the dam were closed in 2003, the environment of marshlands in the middle and lower reaches of the Yangtze River, including Dongting Lake, has been substantially changed, including water level and temperature [26,27]. These changes may influence the distribution of snails [28]; hence, we used the related information from before the Three Gorges Dam functioned in 2003.

*Oncomelania* snail is an amphibious freshwater snail, and its life cycle is closely related to water. The demand of *Oncomelania* snails for water differs according to their growing stages. Water is essential to juvenile snails for growth and development, while adult snails prefer wetland. Our results showed that the number of days inundated with water was significantly related to the occurrence of snails (*p* < 0.05), and that the range of time of habitats inundated water for snail survival was from about 2 months to 7 months, which was similar to some previous estimates that the range is from 3 to 8 months and the optimum is 5 or 6 months [29]. The number of days inundated with water is determined by water level and elevation. When the water level is higher than the elevation, a marshland is considered to be flooded. A previous study [6] found that there was a correlation between water level and the density of living snails. The density of living snails in all areas has been decreasing in Hunan Province since the Three Gorges Dam was built in 2003. The relationship between the number of floods per year and the density of living snails was more pronounced in the medium and high elevation areas. The density of living snails kept decreasing from 2003 to 2014; however, in low elevation areas, the density of living snails decreased at first after 2003 and then increased after 2011. These findings suggested that the number of days inundated with water might be a crucial factor that affects the survival and geographical distribution of snails. Both a too long and a too short period inundated with water might result in the death of adult snails or influence the growth and development of young *Oncomelania* snails. The number of days inundated with water might have a different impact on snails in different seasons. “Water in summer and land in winter” is a typical trait of snail habitats in marshland. On one hand, a long period of flooding in winter is not good for the survival of snails and early flooding in spring hinders snails from laying eggs; on the other hand, flooding in late summer counteracts the hatching of snail eggs [21].

Rainfall is an important determinant for the number of days inundated with water. Rainfall has also been shown to impact the distribution and density of *Oncomelania* snails and the snails’ infection rate [30,31]. Our GAM showed a significant relationship between annual rainfall and snail distribution (*Z* = −3.641, *p* < 0.05). Previous studies reported that *Oncomelania* snails were all distributed in regions with annual rainfall of more than 750 mm in China [32]. In our study, the annual rainfall of all the monitoring sites in Hunan Province was above 989 mm. It seems that rainfall is not a key factor determining the geographical distribution of snails in Hunan province. Although our results showed that there was more rainfall in the areas without snails than in the areas with snails, the number of the days inundated with water was significantly lower in the areas without snails than areas with snails. This might be because the elevation of the marshlands varies in different areas in the province. The marshlands in the mountainous southwest (the areas without snails) are steeper compared to the northeast areas (the areas with snails) and the elevation changes rapidly. Since the marshlands in the northeastern part are smooth and flat, although the precipitation in the southwest of Hunan Province was more abundant than in the northeast, the number of days inundated with water was smaller. Moreover, our GAM also showed that the interaction between annual rainfall and lowest air temperature in a year significantly affected the occurrence of snails (*Z* = −3.697, *p* < 0.05). This finding is consistent with a previous study which reported that the survival rate of dry environment snails was higher than that of wet environment snails when temperature was the same. This difference between the survival rate of “dry” and “wet” snails may have resulted from the different ambient conditions. In the “dry” state, the snails are inactive, dehydrated, and their opercula are closed. However, in a “wet” state the snails are active, rehydrated, and their opercula are open [33].

Ambient temperature is another important ecological factor for the growth and development of *Oncomelania* snails and can influence the geographical distributions of uninfected snails and infected snails [34,35,36]. In China, *O. hupensis* is distributed along the Yangtze River basin in lake regions and mountainous areas of the provinces of Sichuan and Yunnan, where the yearly average air temperature is between 16 °C and 18 °C and the lowest air temperature in a year is between −2 °C and −16 °C [33]. Nevertheless, our results showed that the annual average temperatures were above 16 °C in all investigated areas. The results from GAM shows a relationship between lowest air temperature in a year and snail distribution (*Z* = 3.648, *p* < 0.05). This result is consistent with other studies which reported that the extreme low temperature prevents the transmission of *Oncomelania* snails towards the north [36]. However, as shown in Table 1, lowest air temperature in a year was not significantly different between the areas with and without snails (*p* > 0.05). It might be due to a low power for the Whitney U test, or other reasons we do not know. Our study showed that the lowest temperature in a year was above −2.88 °C in all investigated areas, and a temperature between −2 °C and −16 °C is suitable for the survival of snails. Hence, the results indicated that although the temperature is an important factor affecting the distribution of snails, it might not be a key determinant for the geographical distribution of snails in Hunan Province. Our observations also showed that hours of daylight were weakly associated with the occurrence of snails (*Z* = −2.674, *p* < 0.05). However, the range of the hours of daylight between the areas with (117.46–138.72 h) and without (117.03–138.72 h) snails was very close.

Our study has the following limitations. The study did not include some potentially important factors such as soil moisture and land vegetation [37,38]. Instead, we included some factors which are strongly associated with soil moisture (e.g., rainfall and water level) in our study. Although we did not collect data for land vegetation during the study period, the field sites selected are all grasslands. Besides, anthropogenic activities such as mollusciciding and environmental modification are also known to influence the distribution of *Oncomelania* snails, however, these factors were not included in our paper. Further study is necessary to consider the relationship between anthropogenic factors and *Oncomelania* snail distribution.

## 5. Conclusions

The number of days inundated with water might be a key factor determining the geographical distribution of *Oncomelania* snails in Hunan Province. The favorable number of days inundated with water for the survival of snails might range from 2 to 7 months. The study also shows that rainfall and hours of daylight were associated with the distribution of *Oncomelania snails*.

## Figures and Tables

**Table 1:** Minimum, median and maximum of the air temperature, precipitation, days inundated with water, relative humidity and hours of daylight in different areas.

Variable	Areas with Snails	Minimum	Median	Maximum	*Z*-Value	*p*-Value
Annual average temperature (°C)	No	16.82	17.42	18.10	−1.245	0.213
	Yes	17.42	17.61	17.61		
Extreme low air temperature (°C)	No	−2.88	−2.65	−2.10	−1.301	0.193
	Yes	−2.88	−2.34	−2.34		
Annual rainfall (mm)	No Yes	1230.5 989.57	1466.57 1422.00	1647.57 1565.57	−2.757	0.006
Days of daily precipitation greater than 0.1mm	No Yes	12.13 12.13	13.00 12.13	13.00 13.00	−12.962	<0.001
Days inundated with water	No	0.00	51.50	364.00	−6.849	<0.001
	Yes	56.00	126.50	212.00		
Relative humidity (%)	No	77.38	81.12	81.41	−5.053	<0.001
	Yes	77.38	77.38	81.41		
Hours of daylight (H)	No	117.03	117.46	138.72	−7.808	<0.001
	Yes	117.46	138.72	138.72		

**Table 2:** Generalized Additive Model of factors affecting the distribution of snails.

Parameters	Estimate	Standard Error	*Z*-Value	*p*-Value
Hours of daylight	−0.36220	0.13543	−2.674	0.007
Lowest air temperature in a year	1321.47197	362.27676	3.648	<0.001
Annual rainfall	−0.31043	0.08525	−3.641	<0.001
Days of daily rainfall greater than 0.1 mm	−207.74774	56.79148	−3.658	<0.001
Interaction between lowest air temperature in a year and annual rainfall	−0.1181	0.03215	−3.697	<0.001
Interaction between lowest air temperature in a year and days of rainfall greater than 0.1 mm	−87.03137	24.58443	−3.540	<0.001
Approximate significance of smooth terms				
Parameters	Edf ^a^	Ref.df ^b^	χ^2^	*p*-value
Days inundated with water	7.808	7.982	31.28	<0.001

Note: ^a^: Edf = estimated degrees of freedom for the smooth term; ^b^: Ref.df = estimated residual degrees of freedom.
